# Proliferating Giant Pilomatrixoma: A Case Report

**DOI:** 10.7759/cureus.84988

**Published:** 2025-05-28

**Authors:** Liliana Berenice Alcázar García, Zabdy Elienai Frayre García, Alejandra Zahaydé Grijalva Aguilar, Ernesto Velazco Manzo, Mercedes Hernández Torres

**Affiliations:** 1 Dermatology, Instituto Dermatológico de Jalisco “Dr. José Barba Rubio”, Guadalajara, MEX; 2 Surgery, Instituto Dermatológico de Jalisco “Dr. José Barba Rubio”, Guadalajara, MEX; 3 Dermatopathology, Instituto Dermatológico de Jalisco “Dr. José Barba Rubio”, Guadalajara, MEX

**Keywords:** benign adnexal tumor, giant pilomatrixoma, hair follicle matrix tumor, malignant neoplasm mimic, proliferating pilomatrixoma

## Abstract

Pilomatrixoma is a benign adnexal tumor, frequently observed in pediatric and young adult populations, and originates from the hair follicle matrix. While the most common clinical presentation is a subcutaneous nodule, multiple clinicopathological variants have been identified. One such variant is the giant pilomatrixoma, defined by a size of 4 cm or greater, which may mimic malignant neoplasms such as angiosarcoma or amelanotic melanoma in adults. We present the case of a 36-year-old female patient who presented with an exophytic mass on the right cheek, which exhibited rapid growth following trauma. Due to the suspicion of malignancy, an excisional biopsy with a 5 mm margin was performed, and histopathological examination revealed a proliferating pilomatrixoma. This case is noteworthy for presenting two variants: classification by size (giant pilomatrixoma) and histological subtype (proliferative). It is essential to ensure appropriate surgical management, as incomplete excision may lead to local recurrence and/or malignant transformation.

## Introduction

Pilomatrixoma is a benign skin neoplasm, also known as Malherbe's calcifying epithelioma, derived from the hair follicle matrix. It presents as firm, asymptomatic nodules smaller than 3 cm, typically found on the head, neck, and extremities of children and young adults between 5 and 15 years old, with 75% of cases occurring within this age group and a higher prevalence in females. The incidence of pilomatrixoma worldwide is estimated to be approximately 1 in 800 to 1,000 cutaneous tumors, representing 41% of benign neoplasms. Pilomatrixoma accounts for 1%-15% of benign dermatological lesions and has a favorable prognosis. However, proliferating pilomatrixoma is a rare variant that appears as a rapidly growing vascular lesion in elderly patients [1,2]. We present a very particular case of a patient who falls outside the common age range of presentation, with extremely rapid growth and a very favorable response to conventional surgical excision.

## Case presentation

**Figure 1:**
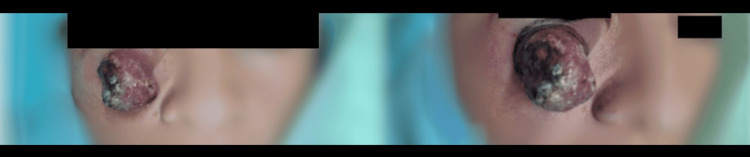
An exophytic neoplasm measuring 4.8 cm × 4.6 cm × 4 cm, erythematous-violaceous in color, with well-defined, regular borders; an irregular surface; and central ulceration, covered by a hemorrhagic crust and abundant keratin.

A 36-year-old female patient presented to the clinic with an exophytic lesion measuring 4.8 cm × 4.6 cm × 4 cm. The lesion was erythematous-violaceous in color, with well-defined borders, an irregular surface, and central ulceration, covered by a hemorrhagic crust and abundant keratin (Figure 1).

**Figure 2:**
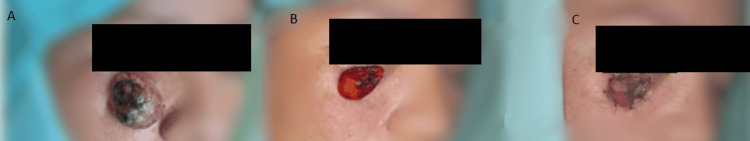
(A) A newly formed tumor-like growth located in the malar region, with unclear etiology; (B) An excisional biopsy was performed, removing the lesion with a 0.5 cm margin of surrounding tissue extending down to the deep subcutaneous layer to ensure adequate sampling for histopathological analysis; (C) The surgical wound was carefully dressed with a hydrocolloid dressing to promote healing and provide a protective barrier against external contaminants.

The patient reported pain upon manipulation and the presence of purulent discharge. Following a seven-day course of antibiotics, there was a slight reduction in lesion size and improvement in local pain. The clinical differential diagnoses included amelanotic melanoma and angiosarcoma. Given the suspicion of malignancy, an excisional biopsy was performed with a 0.5 cm margin extending into the deep subcutaneous tissue, and a hydrocolloid dressing was applied (Figure 2).

**Figure 3:**
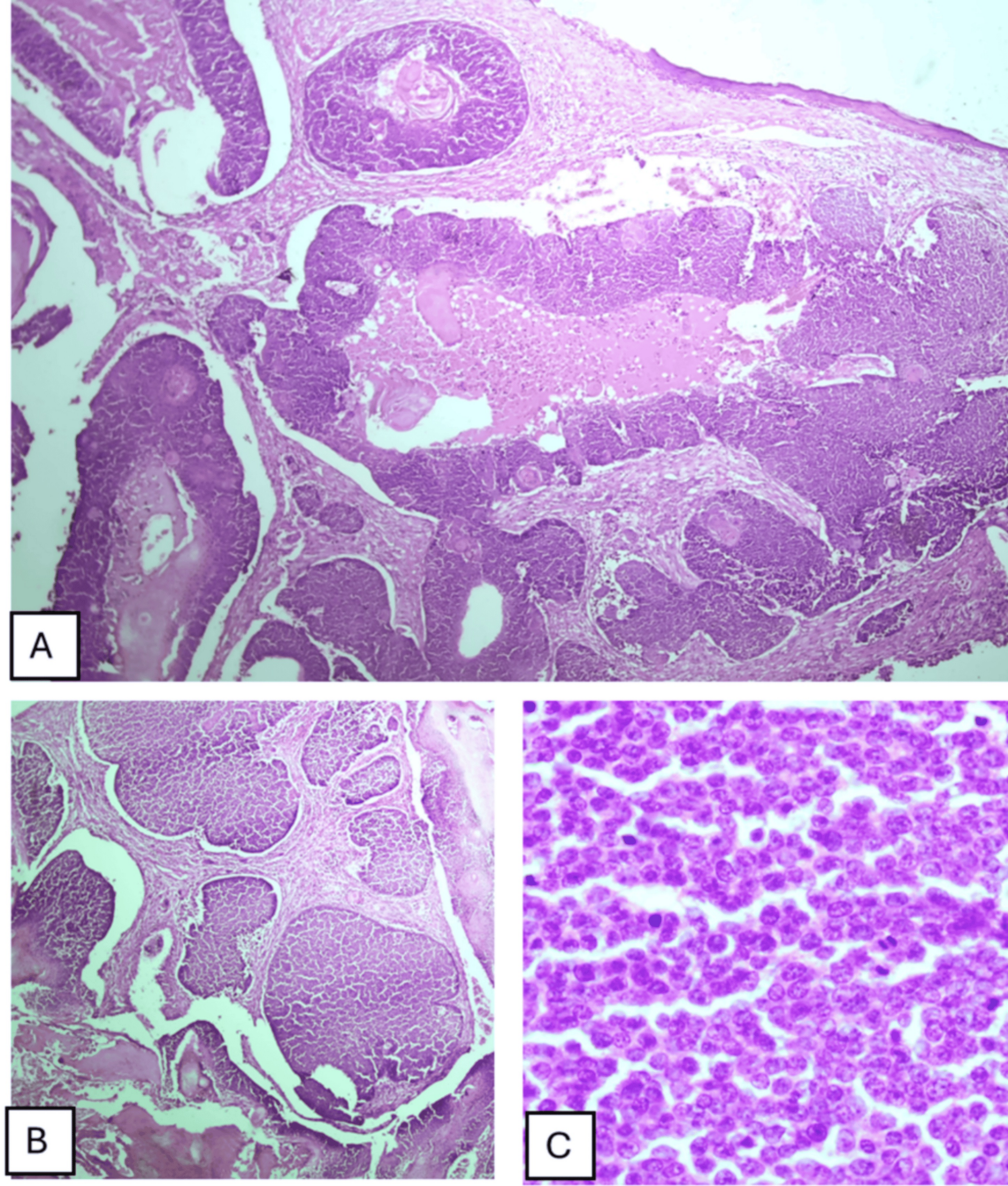
(A)-(B) A proliferation of basaloid epithelial cells infiltrating and extending into the deep dermis, suggesting an aggressive or invasive growth pattern; (C) Presence of follicular matrix cells exhibiting nuclear atypia, characterized by irregular nuclear morphology, along with a mitotic rate of 4-6 mitotic figures per high-power field, indicating active cellular division and potential malignancy.

Histopathological examination revealed a proliferation of basaloid epithelial cells exhibiting matrix keratinization and the formation of ghost cells. Numerous mitotic figures were observed in certain areas, leading to the diagnosis of proliferating pilomatrixoma (Figure 3).

**Figure 4:**
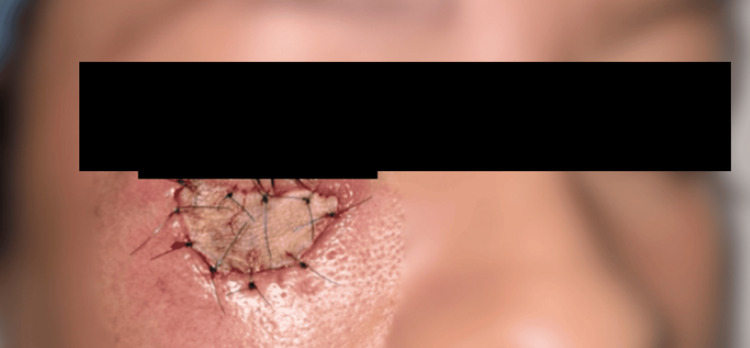
Reconstruction with a full-thickness skin graft. Reconstruction of the surgical defect was performed using a full-thickness skin graft, harvested from a donor site on the patient with similar texture and pigmentation, to ensure optimal aesthetic and functional integration. The graft was meticulously secured in place, ensuring proper adherence and vascularization to promote successful healing.

A complete surgical excision down to the muscular plane was subsequently performed. The surgical specimen was reported as tumor-free, and reconstruction was accomplished using a full-thickness skin graft (Figure 4).

**Figure 5:**
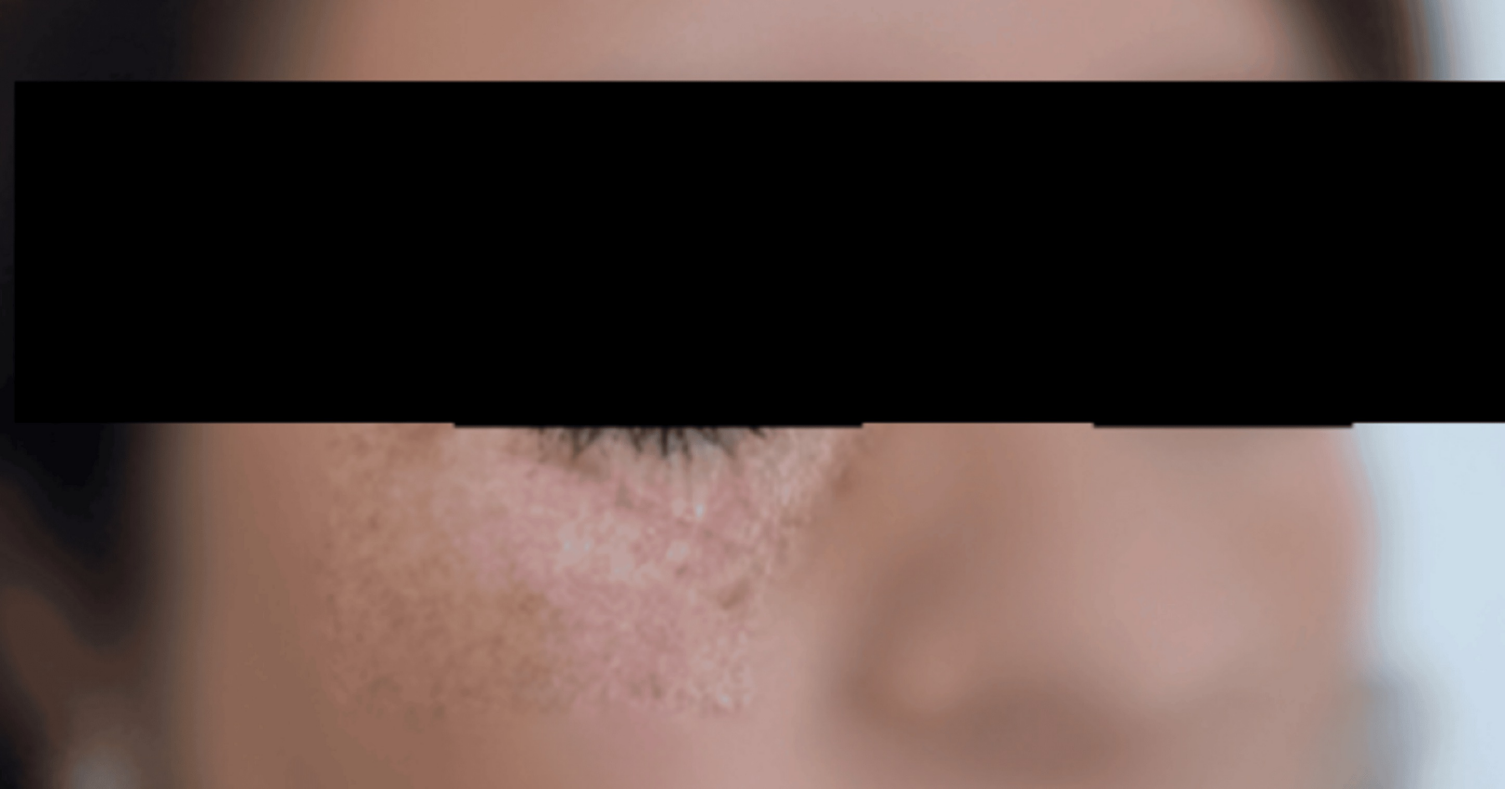
A six-month follow-up evaluation revealed satisfactory functional and aesthetic outcomes, with well-integrated graft healing, minimal scarring, and no signs of recurrence or complications. The patient exhibited good skin texture and contour restoration, with preserved mobility and no functional impairments.

At the six-month follow-up, the patient demonstrated no signs of tumor recurrence and exhibited satisfactory functional and aesthetic outcomes (Figure 5).

## Discussion

Pilomatrixoma (from the Latin pilus, meaning "hair"; matrix, meaning "matrix"; and the Greek suffix -ōma, meaning "result of a process" or "tumor") is a benign neoplasm originating from the follicular matrix cells, with differentiation towards both the hair matrix and cortical cells. In rare instances, it may undergo malignant transformation. First described in 1880 by Malherbe as a calcified epithelioma, the lesion was subsequently redefined in 1961 by Forbis and Helwig as a follicular tumor, and the term pilomatrixoma was coined [1,2].

The incidence of pilomatrixoma worldwide is estimated to be approximately 1 in 800 to 1,000 cutaneous tumors, representing 1%-15% of benign neoplasms. It primarily affects children between the ages of 5 and 15, with 75% of cases occurring within this age group and a higher prevalence in females. In pediatric populations, pilomatrixoma accounts for approximately 10% of all cutaneous tumors. Multiple lesions are rare, occurring in 2%-3.5% of cases, and may be associated with various syndromes, including myotonic dystrophy (Steinert's disease), Gardner syndrome, Turner syndrome, xeroderma pigmentosum, nevoid basal cell carcinoma syndrome, medullary thyroid carcinoma, and Rubinstein-Taybi syndrome [2].

In the literature, up to 2011, 13 patients had been described in four reports of proliferating pilomatricoma, ranging in age from 42 to 88 years (mean age: 66.5 years). However, no further information has been collected after that year. In Mexico, the incidence and prevalence of proliferating pilomatrixoma remain undetermined due to its rare diagnosis. No specific pathophysiological feature has been described to justify the high rate of cell proliferation other than what occurs in classic pilomatrixomas. A review of dermatopathology records at the Instituto Dermatológico de Jalisco “Dr. José Barba Rubio” identified only three cases of proliferating pilomatrixoma, including the present case, over a 13-year period (2011-2024). The mean age at presentation was 68 years, with an average disease duration of 4.8 months. The lesions were localized to the face in two cases and the trunk in one case, with the largest lesion measuring 12 cm in diameter. Notably, a male predominance was observed in this cohort, which contrasts with findings from international reports.

Clinically, pilomatrixomas are heterogeneous lesions composed of follicular matrix cells, which contain varying amounts of hemosiderin, melanin, and calcium. The high calcium content contributes to the lesion’s firm consistency and is responsible for the characteristic "tent sign," wherein the skin folds into angular facets [1,3]. There is ongoing debate regarding whether proliferating pilomatrixoma represents a distinct clinical entity, due to its rapid growth and atypical appearance. Its marked vascularization may mimic malignant tumors [3,4]. Additionally, it typically presents in patients over the age of 70, in contrast to the classic presentation of pilomatrixoma [5].

The classification of pilomatrixoma as "giant" remains a subject of debate, with some authors defining giant lesions as those exceeding 3 cm in diameter, while others set the threshold at 5 cm. This distinction holds clinical significance, as larger lesions have been associated with hyperparathyroidism and hypercalcemia, necessitating laboratory investigations to rule out underlying metabolic disorders [6]. Several clinicopathological variants of pilomatrixoma have been described, including perforating, anetodermic, giant, and atypical forms, as outlined in Table 1 [7].

**Table 1:** Clinicopathological variants. Table credit: [7]

Characteristics	Perforating	Anetodermic	Proliferating	Giant	Atypical
Clinical	Nodular, with erosions on its surface	Appearance similar to a blister, scar, or "empty sac"	Large size and fast growth from 1.5 to 5.5 cm	Size greater than 3 cm, up to 34 cm; they ulcerate; malignant neoplasm mimics	Nodular, large, and firm
Histopathological	The calcifications come out into the epidermis without breaking its integrity	Lymphatic dilation and congestion, disruption of collagen fibrils, and absence of elastic fibers	Large lobular proliferation of basaloid cells, with small foci of ghost cells, and a large number of mitoses (4-15 per high-power field)	Basaloid and ghost cells, multinucleated giant cells, squamous cells, and calcium deposits	Focal infiltrative pattern in the periphery, more than 5 mitoses per high-power field, with necrosis

Histopathologically, proliferating pilomatrixoma exhibits characteristic changes first described by Kaddu et al. in 1997 [5]. These changes include a dermal proliferation of basaloid cells extending into the subcutaneous tissue, with variable nuclear atypia and numerous mitotic figures (ranging from 4 to 15 per high-power field), along with focal areas of eosinophilic cornified material [3,4]. Necrotic areas may also be present; however, there is no evidence of lymphovascular or perineural invasion, which is crucial for distinguishing it from pilomatrical carcinoma [1,7]. Some authors suggest that proliferating pilomatrixoma may serve as a precursor to pilomatrical carcinoma. However, pilomatrical carcinoma presents distinct histopathological features, including poorly defined margins, an infiltrative growth pattern, pleomorphic basaloid cells, and a high mitotic index (>15 mitoses per high-power field) [7].

The differential diagnosis includes pyogenic granuloma, arteriovenous malformations, soft tissue sarcomas, amelanotic melanoma, cutaneous lymphoma, squamous cell carcinoma, basal cell carcinoma with matrical differentiation, and other follicular neoplasms, such as trichoblastoma [8,9].

Surgical excision remains the treatment of choice, with a recommended minimum margin of 3 mm, although there is no international consensus due to the lesion’s rarity. In cases where histopathological findings suggest malignancy, wider margins of 0.5-1 cm are advised, often requiring reconstructive techniques depending on the size of the lesion. Local recurrence has been reported in 0.48% to 6.6% of cases, primarily due to incomplete excision. Close clinical follow-up is recommended due to the potential for transformation into pilomatrical carcinoma. Although there is no exact timeframe for periodic follow-up, with proper excision recurrence is highly unlikely, making the prognosis favorable [9-11].

## Conclusions

In conclusion, we present the case of a patient with proliferating pilomatrixoma, diagnosed through its clinical and histopathological features. The lesion was successfully treated with wide excision, resulting in a favorable functional and aesthetic outcome. This case underscores the importance of diligent clinical monitoring of atypical lesions, due to the potential risk of local recurrence and malignant transformation. As of the most recent follow-up (one year), the patient remains free of recurrence.
